# Pancreatic injury following immune checkpoint inhibitors: A systematic review and meta-analysis

**DOI:** 10.3389/fphar.2022.955701

**Published:** 2022-09-05

**Authors:** Tian Zhang, Yi Wang, Chunhui Shi, Xiaochun Liu, Shangbin Lv, Xin Wang, Weihong Li

**Affiliations:** ^1^ Basic Medical College, Chengdu University of Traditional Chinese Medicine, Chengdu, China; ^2^ Department of Medical Oncology, Baoji Hospital of Traditional Chinese Medicine, Baoji, China

**Keywords:** immune checkpoint inhibitors, immune-related adverse events, meta-analysis, pancreatic injury, pancreatitis, amylase, lipase, randomized clinical trial

## Abstract

**Background:** Pancreatic injury (pancreatitis, amylase/lipase elevation) is a rare adverse event of immune checkpoint inhibitors (ICIs). With the high number of clinical studies on ICIs, the incidence and characteristics of associated pancreatic injury (PI) need to be reevaluated.

**Methods:** A systematic review and meta-analysis was conducted to assess the incidence of PI in cancer patients who received ICIs in randomized controlled trials (RCTs). PubMed, Embase, the ASCO, ESMO, and AACR conference proceedings before 1 April 2022, were investigated for relevant research.

**Results:** 50 RCTs involving 35,223 patients were included. The incidence of ICIs-PI was 2.22% (95% CI = 1.94%–2.53%). The incidence of PI was 3.76% (95% CI = 1.84–7.67%) when combining two ICIs, which was higher than single ICIs [2.25% (95% CI = 1.91–2.65%)]. The ICIs were ranked from high to low based on PI incidence: PD-L1 inhibitors 3.01% (95% CI = 1.86–4.87%), CTLA-4 inhibitors 2.92% (95% CI = 0.99–8.65%) and PD-1 Inhibitor 2% (95% CI = 1.67–2.39%). The ICI with the highest rate of PI was pembrolizumab 7.23.% (95% CI = 1.69–30.89%). In addition, the incidence of severe ICIs-PI was 2.08% (95% CI = 1.76–2.46%); and the incidence of severe PI was 2.32% (95% CI = 1.76–3.06%) when combining two ICIs, which was higher than single ICI [1.95% (95% CI = 1.58–2.41%)]. The ICIs were ranked from high to low according to the incidence of severe PI: PD-L1 inhibitors 3.1% (95% CI = 1.7–5.64%), CTLA-4 inhibitors 2.69% (95% CI = 0.76–9.49%), PD-1 inhibitors 1.80% (95% CI = 1.41–2.29%).

**Conclusion:** Treatment with multiple ICIs result in a higher incidence of PI compared to single ICIs, irrespective of the grade of pancreatic injury. The incidence of PI caused by PD-L1 inhibitors is higher than that of CTLA-4 inhibitors and PD-1 Inhibitor, and Pembrolizumab has the highest rate of ICIs-PI. Although the incidence of ICIs-PI is not high, they are usually severe (≥ grade 3 events).

## Introduction

Since the Food and Drug Administration (FDA) approved Ipilimumab for melanoma treatment in 2011, ICIs have revolutionized the oncology treatment landscape (Vaddepally et al., 2020). Cytotoxic T lymphocyte-associated protein 4 (CTLA-4), programmed death receptor 1 (PD-1) and programmed death ligand 1 (PD-L1) are two of the most widely used immune checkpoint pathways that inhibit T cell immune function at different stages of T cell activation (Zhang et al., 2021). PD-1 and PD-L1 antibody block the PD-1/PD-L1 pathway, enabling upregulation of T cell activation, activating endogenous anti-tumor immune responses, and enhancing their function to kill tumor cells (Alsaab et al., 2017). CTLA-4 inhibitors maintain T-cell activation by deactivating the signal that inhibits T-cell activation and deactivates immunosuppression of regulatory T cells (Treg cells) in the tumor microenvironment (Wolchok and Saenger, 2008). PD-1/PD-L1 and CTLA-4, each with several clinically approved targeted antibodies, are currently used to treat more than 17 cancers (de Miguel Calvo, 2020) and excel in controlling disease progression, prolonging survival, and improving quality of life (Tartarone et al., 2019; Nishijima et al., 2019; Liu et al., 2020). Notably, ICIs achieve long-term efficacy even after treatment discontinuation and are generally better tolerated than other oncology treatments (Kroemer and Zitvogel, 2021). However, ICIs are only effective in some patients and are prone to drug resistance (Kaushik et al., 2022; Chen et al., 2022; Tong et al., 2021). Furthermore, most patients treated with ICIs develop immune-related adverse events (irAEs) (Bertrand et al., 2015; Wang et al., 2019; Xu et al., 2018), some of which can be life-threatening (Bagchi et al., 2021; Takamoto et al., 2022; Maeda et al., 2022; Wang et al., 2018). Since ICIs function by slowing down the immune activation process, they tend to exert off-target effects, including inflammation of different organs or tissues (Robert, 2020). In the digestive system, irAEs occur in the colon, intestines, liver, and pancreas (most common to least common) (Macovei Oprescu et al., 2021; Porcu et al., 2020). Pancreatic injury caused by ICIs (ICIs- PI) mainly refers to pancreatitis and increased lipase/amylase following ICI treatment, and has a relatively low incidence (1.05–7%) (Michot et al., 2018; George et al., 2019; Su et al., 2018; Zhou et al., 2021). However, it may result in chronic pancreatic atrophy, recurrent ICIs-PI, and other adverse outcomes, including death. (Abu-Sbeih et al., 2019; Johnson et al., 2022; Das et al., 2020; Ueno et al., 2021; Jiang et al., 2018).

Although ICIs-PI is uncommon, a growing number of clinical studies related to ICIs have been performed, and its impact on patients’ quality of life and treatment process cannot be ignored. Therefore, it is necessary to update the understanding of the incidence of ICIs-PI, paying particular attention to its mechanism, immunotherapy toxicity, and its clinical management.

This systematic review and meta-analysis focus on the incidence of PI caused by ICIs in the treatment of solid malignancies. The article is based on the latest prospective clinical trials and reports the incidence of PI induced by various ICIs as single agents or in combination with other drugs.

## Materials and methods

This meta-analysis was based entirely on the preferred reporting items for systematic reviews and meta-analysis (PRISMA) (Liberati et al., 2009), and was registered in the International prospective register of systematic reviews (PROSPERO) (CRD42022332230).

### Data sources and searches

A comprehensive search of PubMed and Embase was conducted. Proceedings of the American Society of Clinical Oncology (ASCO), European Society of Medical Oncology (ESMO), and American Association for Cancer Research (AACR) were searched to supplement this study. Literature published up to 1 April 2022 were screened. Three researchers (TZ, YW, and CHS) independently reviewed the titles and abstracts of the studies identified in the search and excluded those not relevant. The full and supplementary texts of the remaining studies were reviewed to determine whether they contained the necessary information. Conflicts in study selection were resolved by referencing the original article and reaching a consensus with the Senior Investigator (WHL). The search strategy can be found at Supplementary Material S1.

### Inclusion and Exclusion Criteria

Inclusion criteria: 1) Patients pathologically diagnosed with solid malignant tumors; 2) Randomized controlled trials (RCTs); 3) The experimental group was treated with PD-1/PD-L1/CTLA-4 inhibitors (Nivolumab, Pembrolizumab, Atezolizumab, Durvalumab, Avelumab, Sintilimab, Cemiplimab, Ipilimumab, Toripalimab, Camrelizumab, Dostalimab, Tislelizumab, Tremelimumab, and Lambrolizumab), with or without other treatments; 4) Control groups were given non- ICI treatments or placebos; 5) Adverse events related to pancreatic injury were fully described; 6) More than 50 patients were involved in each arm.

Exclusion criteria: 1) Hematologic malignancies; 2)Single-arm trials; 3) Phase I clinical trials; 4) Lack of crucial data from trials; 5) Non-English articles; 6) Publication as letters, conference reports, editorials, case reports, animal studies, basic studies, or systematic reviews.

### Data Extraction

For each eligible study, the following variables were independently extracted by 3 different investigators (TZ, YW, XCL): first author, year of publication, study name, tumor type, trial phase, tumor staging, drug class, drug name, drug dose, implementation plan, control group implementation content, and plan, version of the (Common Terminology Criteria for Adverse Events (CTCAE), 2017) number of PI and its incidence, observational indicators. The three authors completed the process independently. In case of discrepancies, the original literature was reviewed and discussed jointly to reach an agreement. If the same trial included multiple publications, the publication with the longest follow-up period or the most informative data was included. Adverse events in clinical trials were reported and graded using The National Cancer Institute’s CTCAE v5.0 (2017). CTCAE ranges from grades 1 to 5, with grades 3–5 AEs considered severe adverse events (SAEs).

### Risk-Of-Bias Assessment

The quality of the individual studies was evaluated according to The Critical Appraisal Skills Program (CASP) (2015). Publication bias was assessed using the Egger test, where *p*-value <0.10 was considered significant bias and further corrected by trim-and-fill method.

### Data Synthesis and Statistical Analysis

The primary objective was to assess the relationship between ICIs and PI compared to other treatments or placebo. The secondary objectives were to compare the PI risks associated with single-ICI versus dual-ICIs (immunotherapy combinations). The PI risks of different categories of ICIs and the PI risks related to various drugs were also compared. Therefore, subgroup analyses were performed on ICI or dual ICIs, ICIs categories, and ICI drugs. The inconsistency index (I2) was used to assess the heterogeneity between studies, where < 30%, 30%–59%, 60%–75%, and > 75% indicated low, medium, high, and considerable heterogeneity, respectively. A fixed-effects model was used if the heterogeneity was <60%; otherwise, a random-effects model was used. The 95% confidence interval (95% CI) and the summation ratios (ORs) for ICIs-PI incidence were calculated using a generalized linear mixed model (GLMM) (Stijnen et al., 2010). All statistical analyses were carried out using R software (version 4.1.3).

## Results

### Literature review and characteristics of included studies

**FIGURE 1:**
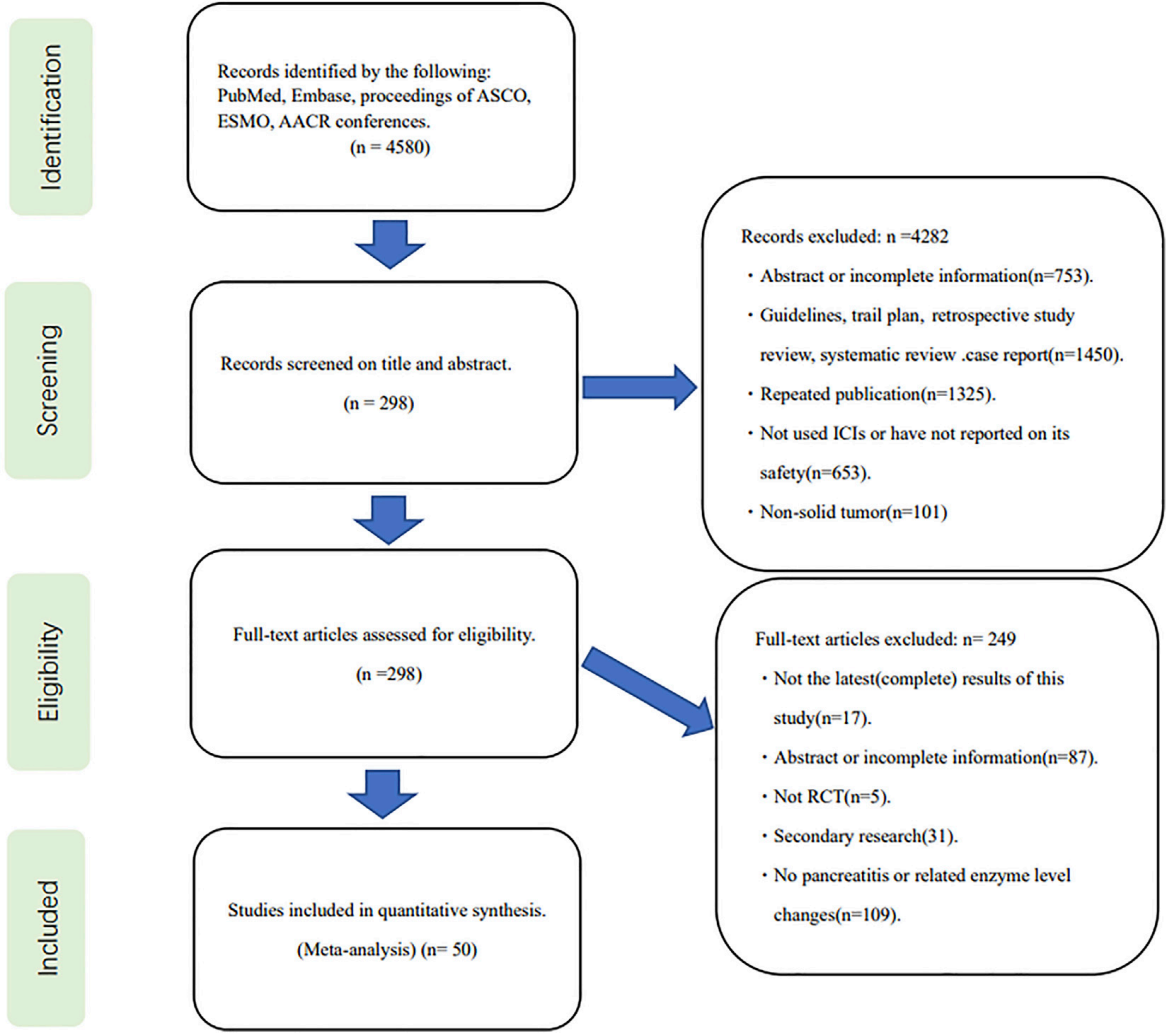
Flowchart for study selection.

The initial search yielded a total of 4580 relevant publications. After screening and eligibility assessment, 50 clinical trials, including 35,223 patients with malignant tumors, were selected for inclusion in this meta-analysis (Figure 1) (Baas et al., 2021; Choueiri et al., 2021; Fennell et al., 2021; Gadgeel et al., 2020; Herbst et al., 2021; Janjigian et al., 2021; Monk et al., 2021; Paz-Ares et al., 2021; Bang et al., 2018; Barlesi et al., 2019; Bellmunt et al., 2021; Brahmer et al., 2015; Burtness et al., 2019; Eggermont et al., 2018; Emens et al., 2020; Eng et al., 2019; Felip et al., 2021; Ferris et al., 2016; Finn et al., 2020; Gianni et al., 2022; Goldman et al., 2021; Gutzmer et al., 2020; Jotte et al., 2020; Lu et al., 2021; Maio et al., 2017; Miles et al., 2021; Moore et al., 2021; Motzer et al., 2020; Motzer et al., 2018; Nishio et al., 2021; O'Byrne et al., 2022; Paz-Ares et al., 2019; Powles et al., 2021; Powles et al., 2020; Powles et al., 2020; Pusztai et al., 2021; Reardon et al., 2020; Reck et al., 2021; Ren et al., 2021; Ribas et al., 2013; Rizvi et al., 2020; Rodríguez-Abreu et al., 2021; Schmid et al., 2018; Socinski et al., 2018; Sun et al., 2021; Weber et al., 2015; Yang et al., 2020; Yau et al., 2022; Zimmer et al., 2020; Huang et al., 2020). The characteristics of the included studies were described in Supplementary Table S1.

The studies included 3 Phase II, 1 Phase II/III, and 46 Phase III RCTs. The most common type of tumors were lung cancer (*n* = 18; 36%), melanoma (*n* = 5; 10%), breast cancer (*n* = 5; 10%), and urothelial carcinoma (*n* = 4; 8%). The studies involved 10 immune checkpoint inhibitors (Pembrolizumab, Nivolumab, Durvalumab, Atezolizumab, Sintilimab, Ipilimumab, Avelumab, Tremelimumab, Cemiplimab, Camrelizumab). 46 studies included patients with stage III-IV cancer, and 4 RCTs assessed the effects of ICIs on early and medium-stage cancer. Three RCTs were multi-armed trials (Herbst et al., 2021; Goldman et al., 2021; Rizvi et al., 2020), and the arms that met the criteria were included in this meta-analysis. The included studies were divided into combination therapy (7 cohorts) and single treatment (43 cohorts), including PD-1 inhibitors (33 cohorts), PD-L1 inhibitors (8 cohorts), and CTLA-4 inhibitors (2 cohorts) for OR analysis.

All studies used randomization methods, but 13 studies did not clarify the specific randomization method. 14 studies used blinding, 34 were open-label, one did not mention the use of blinding, and one did not specify single or multiple blinding. A detailed quality assessment of the included literature is presented in Supplementary Table S2.

### Incidence of ICIs-PI

**FIGURE 2:**
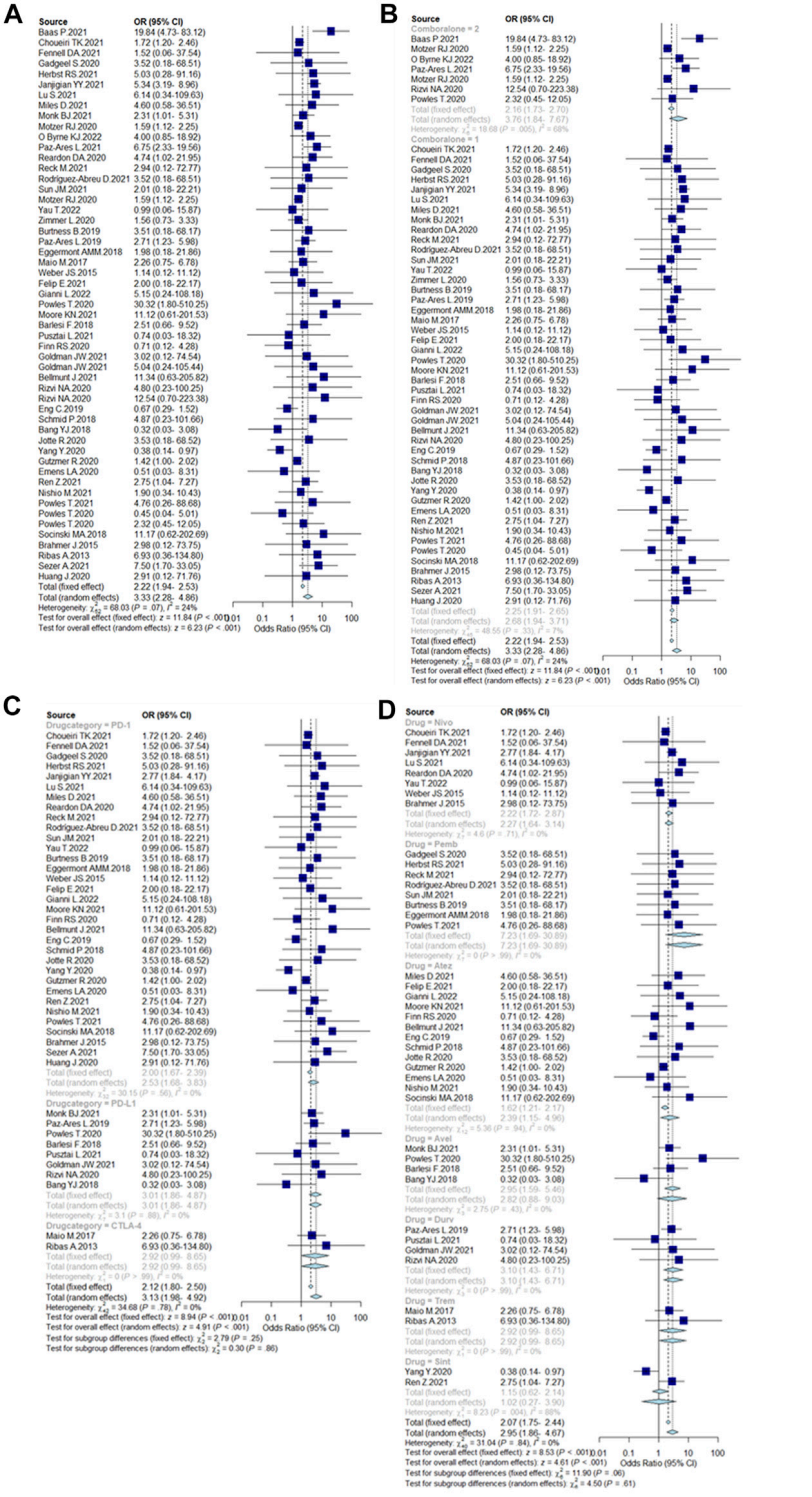
**(A)** Incidence of pancreatic injury caused by ICIs. Incidence rates are represented by boxes and whiskers represent 95% CIs. **(B)** Incidence of pancreatic injury caused by single ICIs or in combination, 2 = dual-ICIs, and 1 = single ICI. **(C)** Incidence of pancreatic injury caused by different drug categories of ICIs. **(D)** Incidence of pancreatic injury caused by various ICI drugs.

The overall incidence of ICIs-PI was 2.22% (95%CI = 1.94–2.53%), and the incidence of PI with dual-ICIs was 3.76% (95%CI = 1.84–7.67%), which was higher than single ICI [2.25% (95%CI = 1.91–2.65%)]. Among the different types of ICIs, the incidence of PI caused by PD-1 inhibitors was 2% (95% CI = 1.67–2.39%), while PD-L1 inhibitors and CTLA-4 inhibitors were similar at 3.01% (95% CI = 1.86–4.87%) and 2.92% (95%CI = 0.99–8.65%), respectively. The incidence of drug-specific PIs were ranked from high to low: 7.23% (95%CI = 1.69–30.89%) for Pembrolizumab, 3.10% (95%CI = 1.43–6.71%) for Durvalumab, 2.95% (95%CI = 1.59–5.46%) for Avelumab, 2.92% (95%CI = 0.99–8.65%) for Tremelimumab, 2.22% (95%CI = 1.72–2.87%) for Nivolumab, 1.62% (95%CI = 1.21–2.17%) for Atezolizumab and, 1.02% (95%CI = 0.27–3.90%) for Sintilimab. Incidence results are summarized in Figure 2.

### Risk-Of-Bias Assessment

**FIGURE 3:**
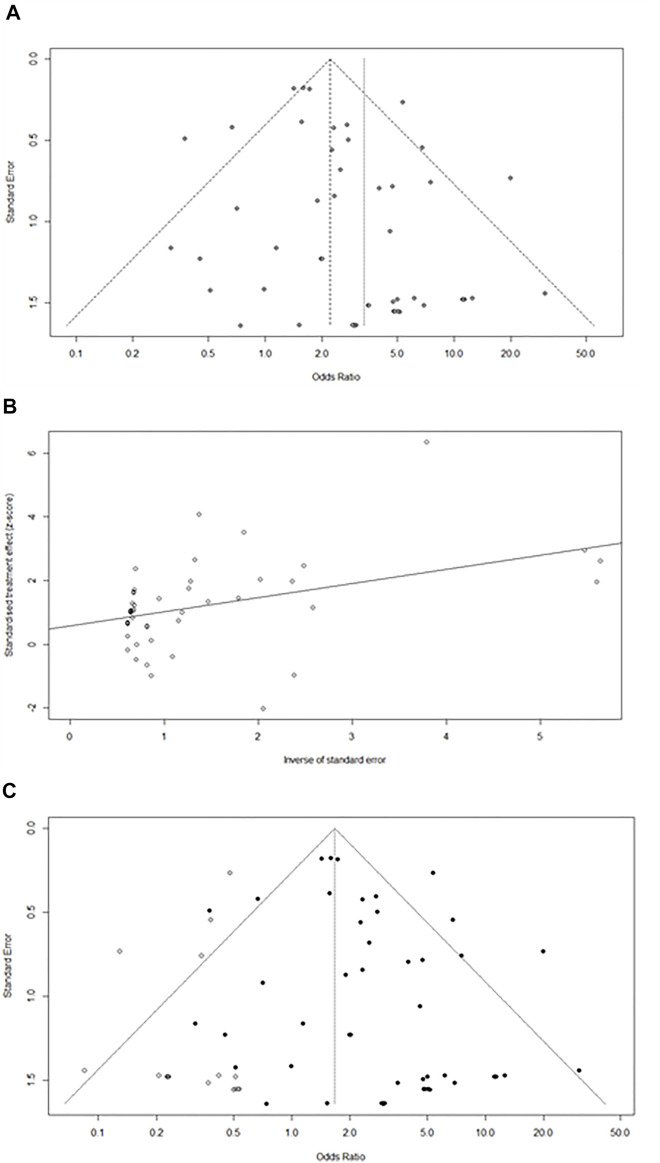
**(A)**Funnel plot of pancreatic injury caused by ICIs.**(B)**Egger test linear analysis plot. **(C)** Publication bias funnel plot after trim-and-fill.

Funnel plots were generated to estimate the intervention effects for each study, and Egger’s test was performed (Figure 3). The linear regression analysis revealed that *p* = 0.02 (*p* < 0.05), indicating a particular deviation in the original data, and 16 studies were missing from the right side of the funnel (k = 69 at this time). The trim-and-fill method was used to test whether publication bias affected the combined effect size. After automatic filling through the algorithm, a new comprehensive effect size of 1.7 (1.2–2.2%) was obtained, which showed no significant difference. Hence, it can be concluded that the combined effect was not significantly affected by publication bias.

### Incidence of severe ICIs-PI

**FIGURE 4:**
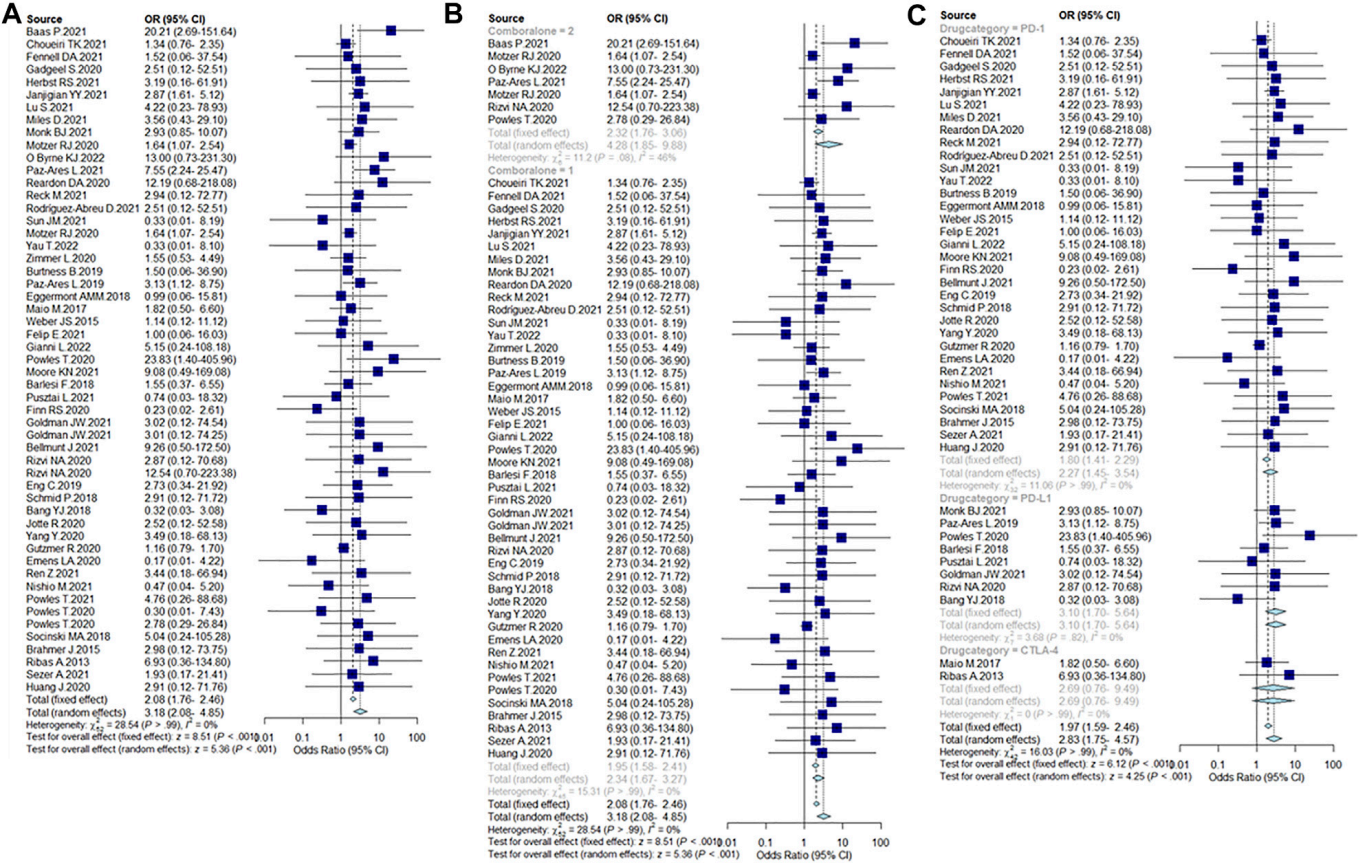
**(A)** Incidence of severe pancreatic injury caused by ICIs. **(B)** Incidence of severe pancreatic injury caused by single ICIs or in combination, 2 = dual-ICIs, and 1 = single ICI. **(C)** Incidence of severe pancreatic injury caused by different drug categories of ICIs.

Compared with non-ICIs treatment, the incidence of severe PI events caused by ICIs was 2.08% (95% CI = 1.76–2.46%). The incidence of severe pancreatic injury events when using a combination of two ICIs was 2.32% (95% CI = 1.76–3.06%), which was higher than single ICIs [1.95% (95% CI = 1.58–2.41%)]. In addition, the incidence of severe PIs caused by PD-1 was 1.80% (95% CI = 1.41–2.29%), PD-L1 was 3.1% (95% CI = 1.7–5.64%), and CTLA-4 was 2.69% (95% CI = 0.76–9.49%). Incidence results are summarized in Figure 4.

## Discussion

Immunotherapy is now a pillar of anti-tumor therapy (Robert, 2020). This study investigated the incidence and characteristics of ICIs-PI by comparing ICIs and non-immunotherapy. In 35,223 patients from 50 RCTs, the all-grades PI incidence was 2.22% (95% CI = 1.94–2.53%), which was less than previous similar studies (George et al., 2019; Su et al., 2018; Zhou et al., 2021). This study aligns with previous studies (George et al., 2019; Su et al., 2018; Zhang et al., 2022; Petrelli et al., 2021) and confirms that the incidence of PI with combined ICI use is significantly higher than that of single ICI. Furthermore, a previous study exploring the safety of more than 2 ICIs suggested that the incidence of elevated lipase was as high as 7.2% (95% CI = 5.2–9.9) (Zhou et al., 2021), which is significantly higher than these results. The analysis of different types of ICIs revealed that PD-L1 inhibitors caused more PI [3.01% (95% CI = 1.86–4.87%)] than CTLA-4 inhibitors [2.92% (95% CI = 0.99–8.65%)] and PD-1 inhibitors [2% (95% CI = 1.67–2.39%)]. The results contradicted the previous perception that CTLA-4 inhibitors are most likely to cause ICIs-PI (Bagchi et al., 2021; George et al., 2019; Su et al., 2018; Kohlmann et al., 2019). In addition, the highest incidence of ICIs-PI was observed with Pembrolizumab, 7.23% (95% CI = 1.69–30.89%), which is in accordance with a previous study (Chen et al., 2021).

The incidence of severe ICIs-PI was 2.08% (95% CI = 1.76–2.46%). As a less common irAE, the incidence of severe PI is slightly lower than the incidence of all-grade PI. It can be assumed that the risk of PI due to immunotherapy is small but severe. Furthermore, the incidence of all-grade PI and severe PI were higher with combined ICIs than with single ICI. Moreover, PD-L1 inhibitors showed a higher incidence of severe PI compared to CTLA-4 inhibitors and PD-1 inhibitors. No cases of death due to ICIs-PI were reported in the included studies.

In the case of patients with NSCLC, combining ICIs with chemotherapy inevitably increases the incidence of serious adverse events (Petrelli et al., 2021). A study investigating irAEs of thoracic malignancies suggested that the incidence of elevated amylase levels in NSCLC patients caused by ICI was 0.6–3% (Remon et al., 2018), which is basically consistent with this study. Some scholars believe that the overall incidence of adverse events is similar between PD-L1 and PD-1 inhibitor-treated NSCLC patient cohorts (Pillai et al., 2018). Conversely, it has been suggested that patients with NSCLC who received PD-1 inhibitors tended to exhibit a higher incidence of organ-specific irAE compared to patients treated with PD-L1 inhibitors, particularly in the digestive system (Sun et al., 2019). We present different sounds, as a digestive system irAEs, in this study (cancer type is not distinguished) pancreatic injury in people using PD-L1 showed a higher susceptibility. Unexpectedly, a meta-analysis of irAEs in patients with urinary cancer found that pancreatitis ranked 15th in combined irAEs, which is not a “little problem” that can be ignored (Wu et al., 2022); Meanwhile, the incidence of pancreatitis in breast cancer patients after ICIs and neoadjuvant chemotherapy was 5.41 (1.02–28.66) (Sternschuss et al., 2021); In addition, in a study investigating irAEs in patients with advanced liver cancer, the incidence of elevated lipase and elevated amylase were 11.6% and 10.3%, respectively (Yuan et al., 2020). However, in patients with advanced gastric cancer or gastroesophageal junction cancer, the incidence of elevated lipase is 0.9 (−0.4–2.2) and the incidence of severe lipase elevation is 0.7 (−0.2–1.6) (Yang et al., 2020), indicating that for patients with advanced gastric cancer or gastroesophageal junction cancer, the incidence of elevated lipase is low but the severity after occurrence is high, which is also consistent with the results of our study. In conclusion, the correlation between ICIs-PI and cancer type needs to be further clarified.

The difference between the results of this study and similar studies can be attributed to two aspects. First, it is believed that the incidence of ICIs-PI is a sparse binary classification variable, and to avoid “continuous correction” of zero events and bias in the results of the meta-analysis of sparse data, a generalized linear model for statistics was chosen. Second, many studies using CTLA-4 inhibitors were not included because they did not meet the inclusion criteria for this study. Small samples and non-RCT studies were also excluded, reducing the heterogeneity of this study to some extent, but also affecting the results.

The infiltration of the pancreas by CD3+T lymphocytes, CD8+T lymphocytes, cytotoxic particle-associated RNA binding protein (TIA1+), granulase B+ and neutrophils is believed to be involved in the mechanism of ICIs-PI. This reaction leads to pancreatic cell damage, pancreatic acinar-duct metaplasia, decreased pancreatic function, and pancreatic atrophy (Di Giacomo et al., 2009; Liu et al., 2021; Hirota et al., 2022). The study found that the co-suppressive immune checkpoint receptors CTLA-4 and PD-1 are closely related to the occurrence of acute pancreatitis (Xie et al., 2021). They participate in acute pancreatitis (AP) immune regulation through the T lymphocyte function (Pan et al., 2017). CTLA-4 can inhibit the proliferation and activity of T lymphocytes by inhibiting the phosphatidyl inositol-3-kinase/protein kinase B signaling pathway, cyclin D3, cyclin-dependent kinase 4/6, and nuclear factor κB (Kubsch et al., 2003; Grohmann et al., 2002). The lack of negative immunosuppressive signals from CTLA-4 in the body may cause a decrease in the threshold of lymphocyte activation, leading to the development of autoimmune diseases. The PD-1/PD-L1 pathway inhibits T cell activation and proliferation, resulting in T cell apoptosis and mediating host immune surveillance evasion (Keir et al., 2008; Boussiotis, 2016). The study found that PD-1 gene knockout may increase pancreatic damage in mice and lead to inflammatory cell infiltration after AP (Xie et al., 2021). PD-1 and PD-L1 may be markers predicting the risk of infectious complications in AP patients (Pan et al., 2017; Yu et al., 2021; Chen et al., 2017), and elevated CTLA-4 and PD-1 expression levels may prevent early pancreatitis (Chuanlin Wu and Bai, 2021). Tissue-resident memory T cells (TRMs) maintain pancreatic immune homeostasis by interacting with resident macrophages and PD-1/PD-L1 inhibitory pathways. In addition, Nivolumab significantly increased IFN-γ, TNF-α, IL-2 levels, and the multifunctional index of pancreatic TRMs by inhibiting the PD-1 pathway, which was associated with pancreatic inflammation (Weisberg et al., 2019). Atezolizumab and lamborizumab increased pancreatic damage and increased serum amylase and lipase levels in AP mouse models (Xie et al., 2021). Overall, ICIs dysregulate the local immune homeostasis of the pancreas by blocking the CTLA-4 and PD-1/PD-L1 pathways, causing pancreatic injury.

In-depth research and clinical management of ICIs-PI is challenging due to its rarity and the other potential causes of elevated serum lipase and amylase (Abu-Sbeih et al., 2019; Hsu et al., 2020). According to the CTCAE classification, three aspects are involved in assessing pancreatic injury: pancreatitis, lipase levels, and amylase levels (Table1). Elevated pancreatin levels are often observed after ICI treatment but the patients are asymptomatic and exhibit no imaging abnormalities. Many early clinical trials did not report asymptomatic elevated amylase/lipase because the relationship between asymptomatic elevated amylase/lipase levels and pancreatitis is unclear (Liu et al., 2021). Studies have found that in patients with immune-related increased lipase, the actual incidence of pancreatitis is 14%, while 86% of the remaining patients are asymptomatic, and immunotherapy can be safely continued (Michot et al., 2018). Therefore, some guidelines do not recommend routine monitoring of pancreatin unless pancreatitis is suspected (Brahmer et al., 2018). On the other hand, some researchers believe that while elevated pancreatin alone does not diagnose pancreatitis, they suggest an increased risk (Ismail and Bhayana, 2017). In order to manage ICIs-PI, the clinical symptoms of pancreatitis should be closely monitored before and after the administration of ICIs. When clinical indications are clear, amylase and lipase levels should be assessed, and abdominal imaging should be considered (There are not necessarily pancreatitis image features). When immune-mediated pancreatitis is diagnosed, steroids or other immunosuppressants (if steroids are contraindicated) should be promptly initiated. The dose should be gradually decreased, accompanied by periodic monitoring for recurrent pancreatitis (Hsu et al., 2020). In general, discontinuation of ICIs and subsequent use of immunosuppressive therapy are not associated with poorer prognosis (Horvat et al., 2015; Skribek et al., 2021). Still, the time cost of discontinuing ICIs and the side effects associated with immunosuppressants should not be underestimated. For asymptomatic patients with elevated pancreatin and radiographic abnormalities, it is recommended to initiate intravenous fluids within 48 h of elevation to reduce the risk of long-term adverse events (Abu-Sbeih et al., 2019).

**TABLE 1:** CTCAE v5.0 classification criteria for pancreatic injury.

	Grade				
Adverse events	1	2	3	4	5
Pancreatitis	-	-Enzyme elevation or radiologic findings only	Severe pain; vomiting; medical intervention indicated (e.g., analgesia, nutritional support)	Life-threatening consequences; urgent intervention indicated	Death
Lipase increased	>ULN - 1.5 × ULN	>1.5–2 × ULN	>2–5 × ULN	>5 × ULN	Death
Amylase increased	>ULN - 1.5 × ULN	>1.5–2 × ULN	>2–5 × ULN	>5 × ULN	Death

There is no difference between CTCAE v4.03 and CTCAE v5.0 in the interpretation of the above three categories. ULN, upper limit of normal value.

Some researchers believe that irAEs are positively correlated with the development of objective response rate, progression-free survival, and overall survival in tumor patients (Hussaini et al., 2021). In a study exploring the relationship between irAEs (including pancreatitis) and the median progression-free survival and overall survival of cancer patients, the researchers found that patients with irAEs had significantly longer progression-free survival and overall survival than patients without irAEs (Ishihara et al., 2019). However, no evidence was found on the relationship between increased amylase and lipase levels caused by ICIs and the efficacy of anti-tumor therapy. Similarly, no direct study on the relationship between pancreatitis caused by ICIs and patient prognosis has been found. Therefore, ICIs -PIs cannot be simply considered to indicate good prognosis.

Moreover, the study had some shortcomings. The trial group included ICIs combined with other treatments. Hence, this study could not demonstrate the exact effect of ICIs on PI. Furthermore, the median follow-up time for each RCT was different, which affects the frequency of ICIs-PI and increases confounding factors. There was no comparison of the relationship between drug dose, cancer type, and PI. In addition, studies investigating the effects of Torlipalimab, Dostalimab, Tislelizumab, and Lambrolizumab did not meet the inclusion criteria and were excluded. Finally, in March this year, Lymphocyte activation gene-3 (LAG-3) was approved by the FDA as the third immune checkpoint for clinical use after PD-1/PD-L1 and CTLA-4 (Andrews et al., 2022). However, the relevant clinical data were few and incomplete, so they were not included in this study.

Currently, guidelines do not recommend pancreatin monitoring in cancer patients receiving ICIs. Based on the results discussed earlier, the general public should be educated on pancreatic injury caused by ICIs, especially in severe pancreatic injury. Asymptomatic patients with ICIs-PI also require medical intervention to reduce the likelihood of adverse long-term outcomes. Clinicians should be aware of the challenges related to ICIs-PIs and, if necessary, conduct rational interventions to prevent other complications.

## Conclusion

This study reevaluates the incidence of ICIs-PI. Combined ICIs lead to a significantly higher incidence of PI than single ICI. The incidence of ICIs-PI caused by PD-L1 inhibitors was higher than those of CTLA-4 inhibitors and PD-1 inhibitors, regardless of the adverse event grade. Pembrolizumab was most likely agent to cause ICIs-PI. Furthermore, the incidence of severe PI caused by ICIs is slightly lower than that of all-grade pancreatic injury. It can be concluded that the risk of pancreatic damage with immunotherapy is not high but is usually severe (≥ grade 3).
