# Supplementary material for: Pancreatic injury following immune checkpoint inhibitors: A systematic review and meta-analysis
Source: Front Pharmacol. 2022 Sep 5;13:955701. doi: 10.3389/fphar.2022.955701 (PMC9483178; doi:10.3389/fphar.2022.955701)
Supplement: Supplementary file 2 [file Presentation1.pdf]

PubMed		
	Search Strategy	Results
#6	Search: ((((((((((((Neoplasms) OR Neoplasia) OR Neoplasias) OR Neoplasm) OR Tumors) OR Tumor) OR Malignancy) OR Malignancies) OR Cancer) OR Cancers AND (humans[Filter])) AND (((((((((nivolumab) OR MDX-1106) OR ONO-4538) OR BMS-936558) OR Opdivo)) OR ((((pembrolizumab) OR lambrolizumab) OR Keytruda) OR MK-3475))) OR (((Durvalumab) OR (((((((atezolizumab) OR anti-PDL1) OR immunoglobulin g1,anti (human cd antigens cd274) (human monoclonal mdpl3280a heavy chain) , disulfide with human monoclonal mdpl3280a kappa-chain, dimer) OR MPDL3280A) OR tecentriq) OR RG7446) OR RG-7446)) OR ((avelumab) OR MSB0010718C))) OR ((((((((((tremelimumab) OR ticilimumab) OR CP 675) OR CP675 cpd) OR CP-675) OR CP-675,206) OR CP-675206) OR CP675206) OR CP 675206)) OR ((((((((((((((((Ipilimumab) OR Anti CTLA 4 MAb Ipilimumab) OR Anti-CTLA-4 MAb Ipilimumab) OR Ipilimumab, Anti-CTLA-4 MAb) OR Yervoy) OR MDX010) OR MDX 010) OR MDX-010 M) OR DX-CTLA-4) OR MDX CTLA 4) OR toripalimab) OR Carrelizumab) OR Preliminumab) OR sintilimab) OR Cemiplimab) OR Tislelizumab) OR OR Dostarlimab) AND (humans[Filter]))) AND ((((((((randomized controlled trail) OR controlled clinicaltrail) OR randomized) OR randomly) OR trial)))) AND ((toxicity[Title/Abstract]) OR (safety[Title/Abstract]))) NOT ((Meta[Title]) OR (review[Title])) Sort by: Most Recent	1923
#5	Search: (Meta[Title]) OR (review[Title]) Sort by: Most Recent	675991
#4	Search: (toxicity[Title/Abstract]) OR (safety[Title/Abstract]) Sort by: Most Recent	1,012,240
#3	Search: (((((((randomized controlled trail) OR controlled clinicaltrail) OR randomized) OR randomly) OR trial)) Sort by: Most Recent	2534058

#2	Search: ((((((((nivolumab) OR MDX-1106) OR ONO-4538) OR BMS-936558) OR Opdivo)) OR ((((pembrolizumab) OR lambrolizumab) OR Keytruda) OR MK-3475))) OR (((Durvalumab) OR (((((((atezolizumab) OR anti-PDL1) OR immunoglobulin g1,anti (human cd antigens cd274) (human monoclonal mdpl3280a heavy chain) , disulfide with human monoclonal mdpl3280a kappa-chain, dimer) OR MPDL3280A) OR tecentriq) OR RG7446) OR RG-7446)) OR ((avelumab) OR MSB0010718C))) OR ((((((((((tremelimumab) OR ticilimumab) OR CP 675) OR CP675 cpd) OR CP-675) OR CP-675,206) OR CP-675206) OR CP675206) OR CP 675206)) OR (((((((((((((((Ipilimumab) OR Anti CTLA 4 MAb Ipilimumab) OR Anti-CTLA-4 MAb Ipilimumab) OR Ipilimumab, Anti-CTLA-4 MAb) OR Yervoy) OR MDX010) OR MDX 010) OR MDX-010 M) OR DX-CTLA-4) OR MDX CTLA 4 OR sintilimab) OR toripalimab)OR Carrelizumab) OR Preliminumab) OR Tislelizumab) OR Dostarlimab) Filters: Humans Sort by: Most Recent	17,310
#1	Search: ((((((((Neoplasms) OR Neoplasia) OR Neoplasias) OR Neoplasm) OR Tumors) OR Tumor) OR Malignancy) OR Malignancies) OR Cancer) OR Cancers Filters: Humans Sort by: Most Recent	4148437

Embase

	Search Strategy	Results
#5	#1 AND #2 AND #3 NOT #4	2657
#4	'meta analysis'/exp OR 'meta analysis' OR review:ti OR case:ti	1,792,,009
#3	randomized AND controlled AND ('trial'/exp OR trial) OR (controlled AND trial, AND randomized;) OR 'randomized controlled trial'/exp OR 'randomized controlled trial' OR (pragmatic AND ('clinical'/exp OR clinical) AND trials) OR (randomised AND controlled AND ('study'/exp OR study)) OR (randomised AND controlled AND ('trial'/exp OR trial)) OR (randomized AND controlled AND study;) OR (trial, AND randomized AND controlled)	1,123,423

#2	(('ipilimumab'/exp OR ipilimumab OR (bms AND 734016;) OR bms734016; OR (mdx AND 010) OR (mdxAND 101;) OR 'mdx010'/exp OR mdx) AND 010 OR 'mdx101'/exp OR mdx101 OR strentarga; OR 'yervoy'/exp OR yervoy OR 'ticilimumab'/exp OR ticilimumab OR (cp AND 675, AND 206) OR (cp AND 675206) OR (cp AND 675 AND 206) OR (cp675, AND 206) OR 'cp675206'/exp OR cp675206 OR 'tremelimumab'/exp OR tremelimumab OR 'nivolumab'/exp OR nivolumab OR (bms AND 936558) OR 'bms936558'/exp OR bms936558 OR (mdx AND 1106) OR (ono AND 4538) OR 'm dx1106' OR mdx1106 OR 'ono4538'/exp OR ono4538 OR 'opdivo'/exp OR opdivo OR 'pembrolizumab'/exp OR pembrolizumab OR 'keytruda'/exp OR keytruda OR 'lambrolizumab'/exp OR lambrolizumab OR (mk AND 3475) OR 'mk3475'/exp OR mk3475 OR 'avelumab'/exp OR avelumab OR bavenci OR (msb AND 0010718) OR (msb AND 10682) OR (msb AND 0010682) OR msb0010718c; OR msb) AND 10682; OR 'msb10718c'/exp OR msb10718c OR 'atezolizumab'/exp OR atezolizumab OR Cemiplimab OR 'Cemiplimab'/exp OR (monoclonal AND ('antibody'/exp OR antibody) AND mpdl AND 3280a) OR (monoclonal AND ('antib ody' OR antibody) AND ('mpdl3280a'/exp OR mpdl3280a)) OR (mpdl AND 3280a) OR 'mpdl3280a'/exp OR mpdl3280a OR (rg AND 7446) OR 'rg7446'/exp OR rg7446 OR 'tecentriq'/exp OR tecentriq OR 'tecntriq'/exp OR tecntriq OR 'durvalumab'/exp OR durvalumab OR (medi AND 4736) OR 'medi4736'/exp OR medi4736 OR sintilimab OR toripalimab OR Carrelizumab OR Preliminumab OR Tislelizumab OR Dostarlimab	16,316
#1	acral AND ('tumor'/exp OR tumor) OR 'neoplasms'/exp OR neoplasms OR (acral AND ('tumour'/exp OR tumour)) OR (('neoplasms'/exp OR neoplasms) AND by AND histologic AND type) OR (neoplasms, AND cystic, AND mucinous, AND serous) OR (neoplasms, AND embryonal AND mixed) OR (neoplasms, AND ('germ'/exp OR germ) AND ('cell'/exp OR cell) AND embryonal;) OR (neoplasms, AND glandular AND epithelial;) OR (neoplasms, AND 'hormone dependent;') OR (neoplasms, AND 'post traumatic') OR (neoplastic AND ('disease'/exp OR disease)) OR 'tumor'/exp OR tumor OR 'tumour'/exp OR tumour	6,345,362
